# Defects in Very Long-Chain Fatty Acid Oxidation Presenting as Different Types of Cardiomyopathy

**DOI:** 10.1155/2022/5529355

**Published:** 2022-04-28

**Authors:** Fariba Alaei, Marjan Shakiba, Hedyeh Saneifard, Kourosh Vahidshahi, Mastaneh Alaei

**Affiliations:** ^1^Pediatric Cardiology Department, Mofid Children's Hospital, Faculty of Medicine, Shahid Beheshti University of Medical Sciences, Tehran, Iran; ^2^Pediatric Endocrinology and Metabolism Department, Mofid Children's Hospital, Faculty of Medicine, Shahid Beheshti University of Medical Sciences, Tehran, Iran; ^3^Pediatric Cardiology, Shahid Modarres Hospital, Faculty of Medicine, Shahid Beheshti University of Medical Sciences, Tehran, Iran; ^4^Blood Transfusion Research Center, High Institute for Research and Education in Transfusion Medicine, Tehran, Iran

## Abstract

Cardiac involvement may accompany various inborn errors of metabolism (IEM) including fatty acid oxidation (FAO) disorders, presenting as rhythm disturbances, conduction abnormalities, cardiomyopathies, pericardial effusion, and sudden cardiac death. FAO disorders are rare mitochondrial diseases with variable organ involvements and clinical presentations. Very long-chain acyl-CoA dehydrogenase deficiency (VLCADD) is a FAO disorder with diverse clinical presentations. We report two VLCADD patients with cardiac involvement and diverse presentations. The first patient represents with cardiogenic shock and dilated cardiomyopathy (DCM) at childhood. The second patient represents with suspicious sepsis at early infancy and hypertrophic cardiomyopathy (HCM) at further evaluation. IEM should be thought of in every individual case with suspicious sepsis or cardiac failure regardless of age or previous history.

## 1. Introduction

Very long-chain acyl-CoA dehydrogenase deficiency (VLCADD) is an autosomal recessive fatty acid beta-oxidation disorder of mitochondrial origin. It involves the initial step of beta oxidation in mitochondrial matrix [1]. Disease is caused by mutations in the *ACADVL* gene (17p13.1) and has an incidence of 1 : 50000-1 : 100000 in newborns [2]. It has different presentations and three phenotypes. Early onset type, usually presents within the first months of life, with hypoglycemia, liver disease, cardiomyopathy (CMP), arrhythmias, and pericardial effusion. Infantile/childhood onset type with a later onset usually presents with hypoglycemia and rarely CMP. Late-onset type detected in older children and young adults usually present with isolated skeletal muscle involvement, myalgia, rhabdomyolysis, and myoglobinuria [3].

Diagnosis is made by tandem mass spectrometry in which blood acylcarnitine profile demonstrates increased C14-C18 (C16:1, C14:2, C14:1, and C18:1). Confirmation is performed by VLCAD activity measurement in the fibroblast or lymphocyte culture or by genetic evaluation revealing two pathogenic mutations in the ACADVL gene [4].

The major cardiac manifestations include arrhythmia, conduction abnormalities, CMP with or without pericardial effusion, and sudden death mainly initiated by fasting, exercise, sickness, and fever [2].

Long-chain fatty acids and carnitine need to be transported across the sarcolemma, as fatty acids are the major source of energy for myocardial cells. Any disturbance in energy production in this disorder results in low availability of glucose (hypoglycemia) and formation of ketone bodies, especially during periods of fasting and sickness. On the other hand, accumulation of toxic metabolites (long-chain fatty acids (LCAD)) is cardiotoxic and can explain CMP following toxic metabolite accumulation [2].

In this study, we report 2 cases of VLCADD with CMP, one presenting at early infancy with hypertrophic CMP and the other presenting at childhood with dilated type. No written consent has been obtained from the patients as there is no patient identifiable data included in this case report.

## 2. Case Report

### 2.1. Patient 1

**Figure 1:**
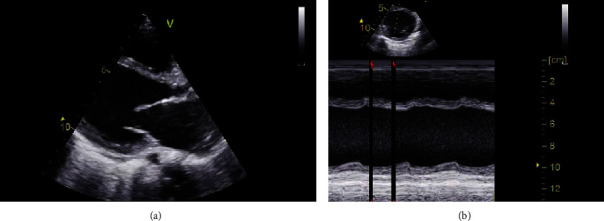
Echocardiographic evaluation at parasternal Log axis (a) and short axis (b) views shows left ventricular dilatation and decreased contractility.

A 4-year and 3-month baby girl was admitted in the ICU with GI symptoms and decreased level of consciousness. She had suffered from abdominal pain, headache, nausea, vomiting, and decreased appetite since 10 days ago. At the time of admission, she was afebrile and hemodynamically unstable with blood pressure of 72/35 mmHg, heart rate of 145/min, weak pulses, and increased capillary refill time. Physical examination revealed grade 3 heart murmurs at left sternal border and apical regions, diffuse expiratory wheezing across the chest, and hepatomegaly. Examination of the abdomen showed no tenderness or guarding. Lab tests demonstrated normal leukocyte count and differential for her age. Acute phase reactants were within normal range. Blood chemistry tests were normal but liver function tests were disrupted (aspartate aminotransferase (AST) = 293 IU/l, alanine aminotransferase (ALT) = 612 IU/L, total bilirubin = 3.48 mg/dl, and direct bilirubin = 1.44 mg/dl). Chest X-ray revealed increased cardiothoracic ratio and pulmonary vascular marking with no significant consolidation. Sinus tachycardia appeared on electrocardiography. Echocardiography showed all chambers dilation, severe mitral, tricuspid and pulmonary regurgitation, reduced ejection fraction (DCM), and mild pulmonary hypertension (Figure 1). She was treated with inotropes and diuretics. Metabolic and rheumatologic evaluation was implemented for evaluation of secondary CMP. Rheumatologic studies were normal. Metabolic work-up revealed normal serum and urine amino acid chromatography as well as normal urine organic acid profile, but increased C14-C14.1-C14.2-C16-C16.1-C18-C18.1 and C18.2 was detected at acylcarnitine profile. Thus, medium chain triglyceride (MCT) oil, low-fat and high-carbohydrate diet was started with the diagnosis of VLCADD.

### 2.2. Patient 2

A 35-day baby girl was admitted with vomiting, poor feeding, poor weight gain, lethargy, and suspicious sepsis. She was the first child of a consanguineous couple, born through an uneventful pregnancy. Anthropometric indices were normal at birth (weight: 2880 g, height: 50 cm, and head circumference: 34 cm). Physical examination revealed heart rate of 185/min and blood pressure of 65/40 mmHg, severe failure to thrive (*W* = 2900 g, height = 50 cm, and head circumference = 34 cm), hypotonia, and hepatomegaly.

During admission, she experienced recurrent episodes of hypoglycemia, so was evaluated for IEM. She appeared normoglycemic when receiving glucose infusion, but hypoglycemia and deterioration of clinical condition recurred repeatedly following restarting of breast feeding and minimizing glucose infusion. Chest radiography demonstrated increased cardiothoracic ratio and echocardiographic evaluation revealed a small ASD with increased left ventricular thickness and normal systolic function in favor of HCM.

Lab tests demonstrated normal complete blood count and electrolytes for age. Uric acid, cholesterol, triglyceride, and creatine kinase (CK) were also within normal limits. Minimal blood glucose level was 30 mg/dl during hypoglycemic episodes, and urine ketone bodies were negative. AST, ALT, and gamma glutamyl transferase (GGT) levels were 146 IU/L, 172 IU/L, and 204 IU/L, respectively. Arterial blood gas exam demonstrated mild metabolic acidosis to normal results during hospitalization. Plasma amino acids profile, serum ammonia and lactate levels, urine carbohydrate chromatography, and galactose 1-phosphate uridyl transferase activity were within normal limits.

Acyl carnitine profile showed specific feature compatible with VLCAD or carnitine palmitoyl transferase 2 deficiency (CPT-2) (C12: 0.64 *μ*mol/l (<0.55), C14: 2.72 *μ*mol/l (<0.5), C14:1: 2.23 *μ*mol/l (<0.25), C14:2: 0.24 *μ*mol/l (<0.2), C16: 16.48 *μ*mol/l (<7) C18: 4.97 *μ*mol/l (<2.5), C18:1: 6.33 *μ*mol/l (<3), and C18:2: 0.73 *μ*mol/l (<0.7)); thus, MCT-based formula was started. This resulted in a dramatic response. Glucose infusion was tapered, and the patient was discharged. During follow-up, she had gained weight, and her height and head circumference had reached normal values. Further cultured fibroblast enzyme activity demonstrated decreased VLCAD activity (CPT2 = 8 (8.5-17.3), VLCAD < 0.18 (1.48-5.2), and Sanger sequencing revealed splicing defect mutation c1269+1G>A in VLFAD gene, both tests confirming VLCADD.

## 3. Discussion

CMPs are primary disorders of myocardium with structural remodeling and resultant ventricular dysfunction. In this regard, a variety of disease classifications has been developed according to the clinical presentation, genetics, and natural history. The main CMP categories include dilated, hypertrophic, restrictive, arrhythmogenic right ventricular, left ventricular noncompaction, and unclassified. CMPs may be primary or secondary to other disorders such as infectious, genetic, toxic, metabolic, endocrine, hematologic, neoplastic, nutritional, neuromuscular, and inflammatory conditions [5, 6].

IEM are consequences of absence or abnormal levels of specific enzymes or enzyme cofactors, resulting in accumulation or deficiency of particular metabolites, mostly inherited as autosomal recessive traits. CMPs may be seen in various IEM and are typically related to energy metabolism impairment or substance storage due to conditions such as glycogen storage diseases, mucopolysaccharidoses, FAO disorders, organic acidemias, and mitochondrial disorders. Newborn screening was offered in all states of United State of America, specific parts of Europe, Australia, and Canada to identify treatable inborn error of metabolism including FAO. Not many developing countries in Asia and Middle East have newborn screening. Japan and China are Asian countries with long experience in newborn screening. Qatar and Saudi Arabia are Middle East countries which started newborn screening during recent years. Newborn screening was started in Iran since 2019. Newborn screening was initiated in a limited area of Iran and is going to cover all parts of the country.

FAO disorders including defects of beta-oxidation enzymes, carnitine deficiency syndromes, and fatty acid transport defects are rare causes of CMP in children. In these disorders, free fatty acids are not metabolized and are stored in the cytoplasm as triglycerides, resulting in myopathy, CMP, and fatty liver [7].

Treatment should include energy supplying to prevent catabolic state, avoiding fasting, and regulating nutritional fat, restricting LCAD consumption and providing MCT supplementation to minimize production of abnormal fatty acid metabolites, by-pass the enzymatic block in *β*-oxidation, and providing ketone bodies as appropriate sources of energy [8].

Therefore, it is important to consider curable genetic conditions, including FAO disorders such as VLCADD, as potential causes of infantile or delayed onset CMPs. VLCADD encompasses a continuum of severity, including minimal symptoms to life-threatening conditions in different age groups, resulting in a very heterogeneous clinical outcome.

There are some case reports discussing cardiac involvement in VLCADD with variety of manifestations presenting in Table 1 [9–11].

Mathur and colleagues studied 37 patients suspicious of FAO disorders and identified 18 genetically documented VLCADD. Among them, 12 (67%) presented with CMP (11 DCM and 1 HCM), in infancy. Finally, 8 survived and 6 gained normal systolic function [12].

Tucci and colleagues performed cardiac function evaluation in VLCADD mice applying magnetic resonance imaging (MRI) and magnetic resonance spectroscopy (MRS). Cardiac dysfunction and dilatation developed during 1-year follow-up despite MCT supplementation. They concluded that cardiac function deterioration was due to specific MCT regimen and as the result of its induced ketogenic state, inhibition of glucose oxidation and transforming of MCT into long-chain fatty acids (LCT) [13].

There are few reports of VLCADD presenting with CMP in the literature. Most cases have presented at neonatal period or early infancy. Our first case demonstrated the disorder at age of 4 years with dilated CMP and no previous history of myopathy or cardiac disorder. The second case presented at early infancy with hypoglycemia and hypertrophic CMP. It seems that early detection and prompt treatment implementation may be lifesaving in this group of patients.

## Figures and Tables

**Table 1:** VLCADD patients' natural history.

Study	Age at presentation	Clinical manifestation	Cardiac presentation	Diagnosis	Outcome
Dereddy NR	2 months	Respiratory distress, failure to thrive, hepatomegaly, hypotonia	Left ventricular hypertrophy and dilatation, reduced systolic function and pericardial effusion	Skin fibroblasts enzyme activity	Normal cardiac function after 8 years follow-up
	4 months	Metabolic acidosis, cardiogenic shock	Left ventricular dilatation with reduced systolic function	Gene mutation	Acceptable cardiac size and function after 4 years follow-up
Katz S	2 days	Lethargy, increased CK and liver transaminases	First: small ASD After 6 months: Massive pericardial effusion, left ventricular hypertrophy and systolic dysfunction requiring ECMO	Plasma acyl carnitine profile, gene mutation	Death
Mathur A^∗^	1day to 22 months	a variety of presentations	Dilated and hypertrophic cardiomyopathy	Gene mutation	Normal systolic function in 6
Sharef SW	Immediate neonatal period	Neonatal hypoglycemia, positive family history	Dilated and hypertrophic left ventricle, reduced ejection fraction, pericardial effusion	Acylcarnitine profile, skin fibroblast enzyme activity	Normal cardiac function after 4 years follow-up

^∗^This study consisted of 18 genetically documented VLCADD cases.
